# Selective Vestibular Neurectomy through the Presigmoid Retrolabyrinthine Approach in the Treatment of Meniere’s Disease

**DOI:** 10.3390/brainsci14040369

**Published:** 2024-04-11

**Authors:** Fabrizio Salvinelli, Francesca Bonifacio, Mara Capece, Denis Aiudi, Alessio Iacoangeli, Fabio Greco, Maurizio Gladi, Maurizio Iacoangeli

**Affiliations:** 1 UOC di Otorinolaringoiatria, Fondazione Policlinico Universitario Campus Bio-Medico, 60126 Rome, Italyf.bonifacio@policlinicocampus.it (F.B.);; 2 Clinica Universitaria di Neurochirurgia, Università Politecnica delle Marche, Azienda Ospedaliero Universitaria delle Marche, 60126 Ancona, Italy; mara.capece@ospedalesancarlo.it (M.C.); s1102195@pm.univpm.it (A.I.); maurizio.gladi@ospedaliriuniti.marche.it (M.G.); maurizio.iacoangeli@ospedaliriuniti.marche.it (M.I.); 3 Istituto di Ricovero e Cura a Carattere Scientifico—Istituto Nazionale di Ricovero e Cura per Anziani (IRCCS—INRCA), 60127 Ancona, Italy

**Keywords:** Meniere’s disease, vestibular neurectomy, presigmoid retrolabyrinthine, extradural endolymphatic sac surgery, combined micro-endoscopic surgery

## Abstract

Background: Meniere’s disease (MD) is a disabling disease, especially in patients who are refractory to medical therapy. Moreover, selective vestibular neurectomy (VN), in these selected cases, can be considered a surgical alternative which preserves hearing function and facial nerve. Methods: We retrospectively studied 23 patients with MD diagnosis and history of failed extradural endolymphatic sac surgery (ELSS) who underwent combined micro-endoscopic selective VN, between January 2019 and August 2023, via a presigmoid retrolabyrinthine approach. All patients were stratified according to clinical features, assessing preoperative and postoperative hearing levels and quality of life. Results: At the maximum present follow-up of 2 years, this procedure is characterized by a low rate of complications and about 90% vertigo control after surgery. No definitive facial palsy or hearing loss was described in this series. One patient required reintervention for a CSF fistula. Statistically significant (*p* = 0.001) difference was found between the preoperative and the postoperative performance in terms of physical, functional, and emotive scales assessed via the DHI questionnaire. Conclusions: Selective VN via a presigmoid retrolabyrinthine approach is a safe procedure for intractable vertigo associated with MD, when residual hearing function still exists. The use of the endoscope and intraoperative neuromonitoring guaranteed a precise result, saving the cochlear fibers and facial nerve. The approach for VN is a familiar procedure to the otolaryngologist, as is lateral skull base anatomy to the neurosurgeon; therefore, the best results are obtained with multidisciplinary teamwork.

## 1. Introduction

Meniere’s disease is an inner ear chronic illness characterized by intermittent episodes of vertigo lasting from minutes to hours, with unilateral fluctuating sensorineural hearing loss, tinnitus, and aural fullness. Its etiology remains unknown, but endolymphatic hydrops seems to be its pathological substrate. The onset of this disease is typically in adulthood, with a peak of incidence between the age of 40 and 60, and it is more frequent in women [1,2]. The aim of the treatment is to reduce the frequency and the severity of vertigo episodes, with a minimal hearing impairment function. Medical treatment includes the use of diuretics (hydrochlorothiazide most commonly) and Betahistine. If medical treatment fails, the second line is represented by an intratympanic injection of corticosteroids (dexamethasone first). The third step is extradural endolymphatic sac surgery (ELSS), as a conservative surgical treatment, followed by an intratympanic injection of gentamicin (ITG). Ultimately, labyrinthectomy and selective vestibular neurectomy (VN) are indicated for refractory cases. VN is chosen in patients suffering from intractable vertigo with a serviceable hearing function and monolateral disease. This technique, performed using different surgical approaches, guarantees a higher vertigo control rate compared to ITG [3,4,5,6]. The background of this research finds its basis on the concept that a destructive surgical treatment like labyrinthectomy is less used nowadays in favor of vestibular nerve section. In our preliminary experience, we took advantage of the presigmoid retrolabyrinthine approach, which brings benefits such as lower morbidity due to hearing preservation and low risk of facial nerve injury. When changing a patient’s lifestyle and conservative first-line medical treatment, such as diuretics and Betahistine, failed together with second- (intratympanic injection of steroids) and third- (ELSS and ITC) line therapies, we suggested our patients to undergo vestibular nerve section.

The aim of this study was to evaluate the effectiveness of surgical selective VN and to contribute to validating this technique, in selected cases, as a therapeutic alternative option in the treatment of patients with refractory MD, with a residual hearing function, after a previous failure of medical and less-invasive surgical treatments.

## 2. Materials and Methods

We retrospectively studied 23 patients with MD diagnosis who underwent VN at the Otorhinolaryngology Department of the “Campus Biomedico” University of Rome (Italy), in collaboration with the Department of Neurosurgery of “Politecnica delle Marche” University, between January 2019 and August 2023.

All patients met the criteria for MD according to the Classification Committee of the Bárány Society and underwent VN after the failure of medical treatments for at least 6 months, including lifestyle modifications (low-sodium diet, good sleep, decreasing stress, no caffeine), and after the ELSS procedure; all analyzed patients were treated according to the quoted scheme, including endolymphatic sac surgery and gentamicin administration. Patients with bilateral disease were excluded as candidates for VN.

All patients were stratified according to their clinical characteristics, assessing the preoperative and the postoperative hearing levels and quality of life. We performed the Dizziness Handicap Inventory (DHI) questionnaire and the Functional Level Scale (FLS—Tabs AAO-HNS 1995) for each patient, both preoperatively and postoperatively, to evaluate the vertigo control. The DHI questionnaire was administered through 25 questions divided into physical, functional, and emotive scales to assess the influence of vertigo on the quality of life. Every patient was asked to define ear fullness and tinnitus as absent, reduced, unchanged, or worsened. The preoperative and the postoperative audiometric examinations were assessed by the bone conduction 4-Pure-Tone Average (PTA), calculating any improvement, worsening, or no change (≤10 dB) according to the AAO-HNS criteria for the hearing level assessment. 

All patients underwent a combined micro-endoscopic selective VN via a presigmoid retrolabyrinthine approach. We used a Storz 0–30 and 45-degree endoscope (Karl Storz, Tuttlingen, Germany) and a Leica surgical microscope (Leica Microsystems, Wetzlar, Germany) for all operations. All surgical procedures were performed by the same medical team (otolaryngologists and neurosurgeons). Intraoperative neurophysiological monitoring and direct stimulation of nervous fibers were always used. Vertigo recurrence and postoperative complication rate (CSF leakage; V, VII, or cochlear nerve damage; intracranial infections) were also considered. 

### 2.1. Operative Technique and Surgical Anatomy

**Figure 1:**
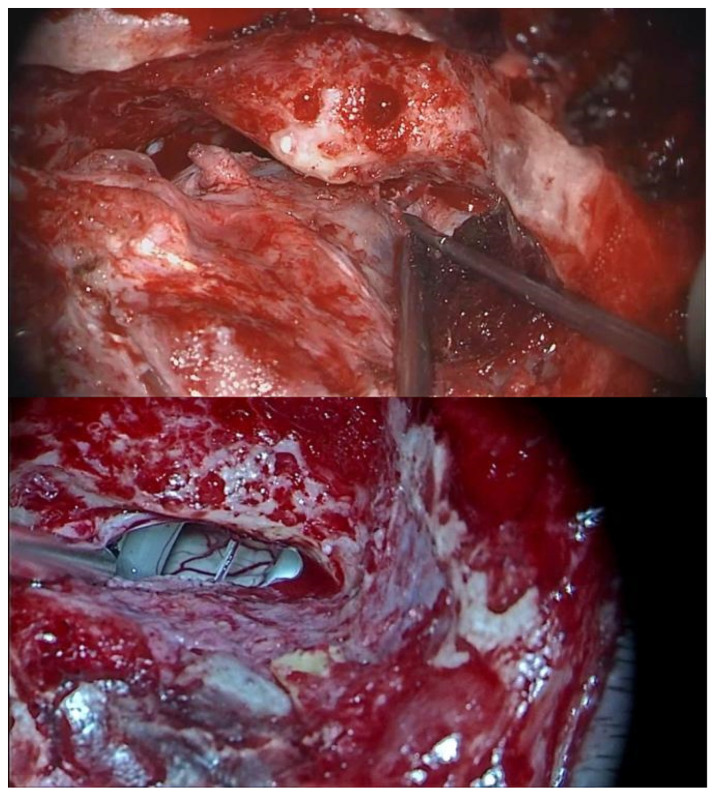
Labyrinthine block exposure. View of the posterior semicircular canal, towards the sigmoid sinus (**upper**); dura mater opening (**lower**) allows an initial anatomical view of the region, i.e., cerebellum, VII–VIII complex, and V nerve.

**Figure 2:**
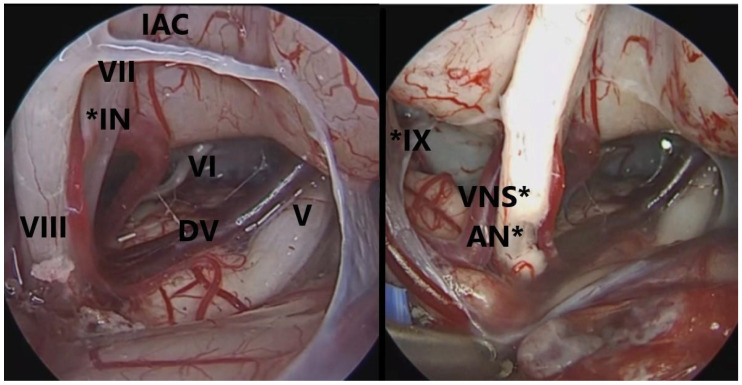
Endoscopic view of the cerebello-pontine angle showing key anatomy (**left**) and vestibular stumps ((**right**), VNS*) after vestibular neurectomy with cochlear nerve preservation (AN*) and showing the close relationships between vestibular nerve and the cochlear and facial nerve. IAC: internal auditory canal; *IN: intermediate nerve; DV: Dandy veins; VNS*: vestibular nerve stump; AN*: auditory or cochlear nerve; *IX: Glossopharyngeal nerve.

A presigmoid retrolabyrinthine approach was performed for all patients. The exposure of the mastoid cortex in these patients showed the signs of previous ELSS. Hence, the following landmarks were exposed: mastoid antrum, petrous sinus, dura mater of the middle, and posterior cranial fossa. The meningeal incision is carried out superior to the endolymphatic sac and posterior to the posterior semicircular canal, with access to the pontocerebellar angle (CPA) (Figure 1). Deliquoration and the opening of the arachnoid is then performed. The use of 0°, 30°, and 45° endoscopes allowed a wide visualization of the CPA, without displacement of the cerebellum, which usually reduces the underlying vision. Once the landmarks (VIII, VII, V, and VI cranial nerves) were recognized, as well as the vascular structures, the authors shifted to the microscope surgical phase. The opening of the cisterns allowed the retraction of the cerebellum, with a larger visual space, and VN was then performed. At this point, the endoscope allowed a correct visualization of the VII cranial nerve, which is usually hidden behind the VIII cranial nerve. By using higher magnification, it is possible to identify the safer site to perform the section of the vestibular nerve, which is usually as proximal as possible to the brain stem, towards the V c.n., since this is the area of further distance from the VII cranial nerve (Figure 2). The authors preferred to dissect the vestibular fibers from the outside to the inside until their elastic retraction allowed the identification of the two vestibular nerve components. It is important that the superior component of the vestibular nerve is moved upwards with suction to verify the complete section of the vestibular nerve from the cochlear nerve with a dissector. In case of even a minimal residual of vestibular nerve fibers, MD symptoms are likely not to resolve. 

This study was conducted in accordance with the Declaration of Helsinki, and ethical review and approval were waived for this study due to its retrospective nature and all the procedures being part of routine standard care.

Informed consent was obtained from all subjects involved in this study and explained the possible inefficacy of the procedure.

### 2.2. Data Analysis

Statistical analysis was conducted using GraphPad Software, 10 and the Shapiro–Wilk test was used to evaluate the normality of the sample. The discontinuous variables were listed with median and interquartile range (IQR). Every variable was analyzed before and after surgery through the Wilcoxon signed-rank test. Statistical significance was determined by *p*-values of <0.05.

## 3. Results

**Figure 3:**
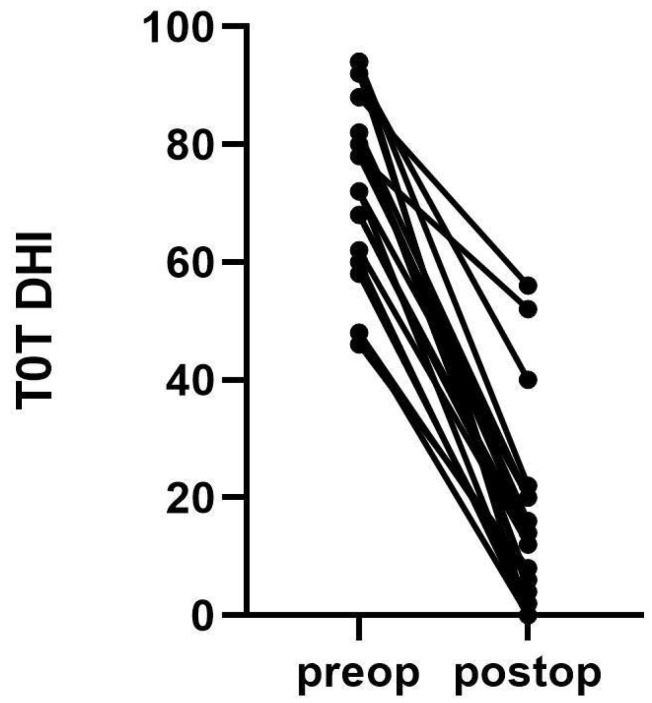
Comparison of Dizziness Handicap Inventory (DHI) scores for each patient before and after surgery.

**Figure 4:**
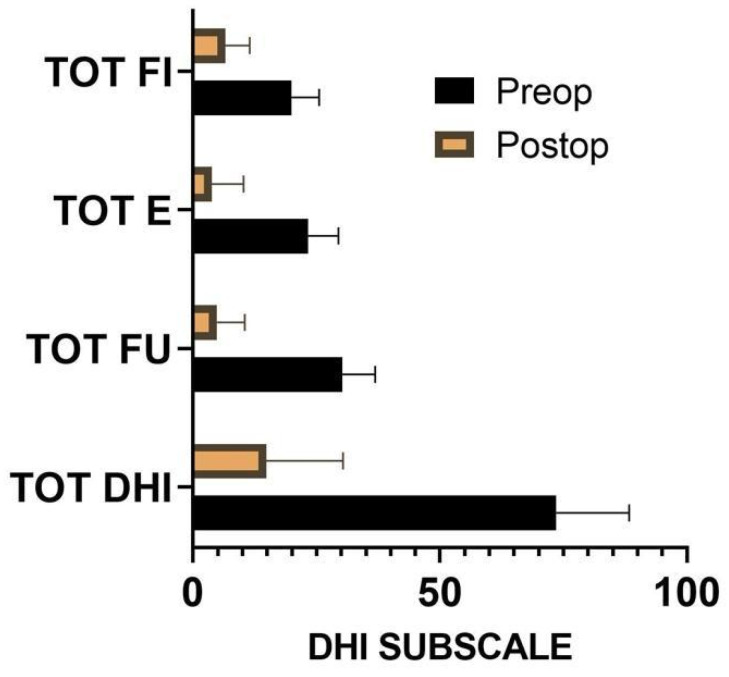
Comparison of average DHI subscale and average total DHI before and after surgery after 12 months of follow-up. DHI, Dizziness Handicap inventory; FI, physical scale; FU, functional scale; E, emotional scale.

**Figure 5:**
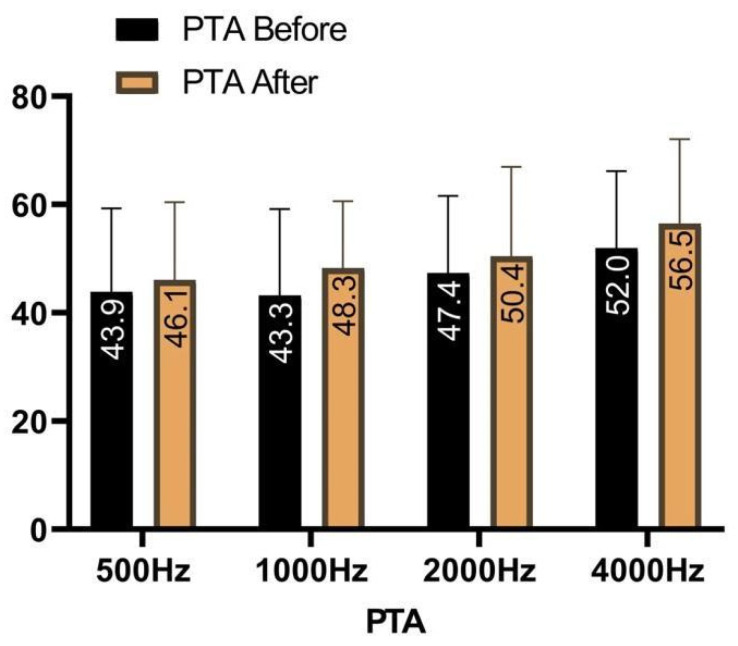
Four-pure-tone average (PTA: 500–1000–2000–4000 Hz) before and after surgery after 12 months of follow-up.

The DHI baseline characteristics of the patients are listed in Figure 3. The Wilcoxon test showed a statistically significant (*p* = 0.001) difference between preoperative and postoperative performance in terms of physical, functional, and emotive scales assessed with the DHI questionnaire (Figure 4). The median was 58 ± 18 IQR, while specific medians were 14 ± 8 for the physical scale, 20 ± 8 for the emotive scale, and 26 ± 6 for the functional scale. The difference before and after surgery was significant also for the FLS (*p* = 0.001), with a preoperative mean of 4.7 ± 0.8 against a postoperative one of 1.6 ± 0.9. The tinnitus was absent after surgery in 4% of the cases, reduced in 35%, unchanged in 57%, and worsened in 4%. Ear fullness was absent after surgery in 30% of the cases, reduced in 57%, unchanged in 13%, and never worsened. In terms of 4-PTA, it was calculated as 46.5 before surgery, against a postoperative 4-PTA of 50.3 (Figure 5). The average hearing loss was 3.8 decibels, resulting in auditory stability (according to the AAO-HNS criteria for hearing level assessment).

No definitive facial palsy or hearing loss was described in this series. One patient required reintervention for a CSF fistula.

In all cases, intraoperative neurophysiological monitoring and direct stimulation of the nervous fibers allowed the selective identification of the V, VII, and VIII cranial nerves.

Thanks to the higher magnification and the better visualization provided by the endoscope, we were able to better identify the vestibular and cochlear components. This also allowed us to appreciate the presence of small vessels and the subtle color differences, which tend to demarcate the two types of nerve fibers.

## 4. Discussion

Selective VN is a potentially valid surgical alternative for patients with refractory MD, with preserved hearing function. The surgical indication, grounded on the international consensus (ICON) on the treatment of MD [3], is given after failure of the two steps of medical therapy (diet and intratympanic corticosteroid) and of the third step, i.e., conservative surgery (ELSS). Hence, according to the ICON, VN is considered a demolition treatment as is ITG, which is a technically simple procedure, but it is not free of possible complications. According to a systematic review, the heterogeneity of data in the literature does not allow us to define the real risk of hearing loss nor the damage risk but they are assessed to be around 26% [7,8]. Zhang, Pullens et al. confirmed this percentage and the insufficiency of the statistical proofs to establish the unique and definitive safety and efficacy of this treatment [7,9]. Yetiser et al. described different studies’ standardization methods regarding the ITG therapeutic scheme, with hearing loss percentages being between 0 and 21% when administered weekly or monthly, confirming the abovementioned heterogeneity of the results [8]. Aminoglycosides and ear toxicity have a well-known association, with instability and ataxia also being other collateral symptoms [10]. ITG [Reviewer 1] seems to damage the efferent fibers, which are probably involved in vestibular compensation [11]. The ITG guarantees an early vertigo control for up to 92.5% patients, with recurrences after 15 years of follow-up occuring in 30% of cases [12,13]. A review comparing vertigo control rates after VN and ITG reported a comparable control rate of vestibular symptoms, with unclear long-term hearing results [11]. Colletti et al. [14] have reported that selective VN has a higher rate of vertigo control with a lower risk of hearing loss than ITG.

The vestibular input to the CNS is normally bilateral and symmetrical, and produces symptoms when it becomes asymmetrical; VN is based on the rationale of eliminating the abnormal vestibular input from the affected side.

The concept of VN was introduced by Charcot in 1874; then, Krause performed the first vestibulocochlear nerve section to treat a patient with severe tinnitus (1898), and Perry performed VN in a patient with MD in 1904. In 1936, Dandy was the first to practice VN via suboccipital craniectomy, sparing hearing function. Later, Silverstein introduced the presigmoid retrolabirynthine approach, followed by the retrosigmoid approach [14,15].

The VIII nerve trunk separates into its double component (cochlear and vestibular) at the internal auditory canal (IAC) level. The identification of the cleavage plane between the cochlear and vestibular components of the VIII nerve, which is distinguished by a fine arteriole and a different coloration (grayish white for the vestibular nerve and pure white for the cochlear nerve), is of paramount importance [11,16].

The middle cranial fossa corridor was one of the most used, but possible complications, such as subdural hematoma and VII cranial nerve paralysis (5–7%) [17,18,19], determined a shift towards different approaches. In addition, the middle fossa approach allows the exposure of the vestibular nerve within the IAC, with a high risk of facial nerve damage. The presigmoid retrolabirynthine approach can be realized through a transcanal or a transmastoid route. All VNs reported in this study were realized, taking advantage of the transmastoid pathway. This approach offers some advantages, such as direct access to the CPA (due to its more lateral trajectory), no need for retraction of the cerebellum, a lower risk of facial nerve damage compared to middle cranial fossa, and a possible better working angle for the vestibular nerve section, which should be as close as possible to the brain stem, towards the V cranial nerve. This approach may offer a direct view of the targeted section area, with clearer anatomic landmarks. On the other hand, it offers a narrow working corridor, with a high potential for injury to the posterior semicircular canal. The retrosigmoid approach guarantees a technically easier craniotomy, provides a clear view of the VIII nerve entering the IAC, due to its more posterior trajectory, and may be combined with a dissection into the IAC if a cleavage plane is not identified at the CPA. However, this approach requires a retraction on the cerebellum and is associated with postoperative chronic headaches [16,20,21]. The retrolabyrinthine and the retrosigmoid pathways share complications, such as CSF fistula. The frequency seems to be higher with the retrolabyrinthine approach, compared to the retrosigmoid approach, where it can be possible to suture the dura. Indeed, in retrolabyrinthine neurectomy, it is not possible to achieve a water-tight closure of the dura mater since the cut of the dura flap is very close to the posterior semicircular canal—in order to gain a better exposure—and the opening is small and deeply located in the mastoid. Therefore, it is preferred to close the surgical cavity with autologous fat. 

Failing to realize a complete vestibular deafferentation can lead to vertigo persistence after surgery. In fact, next to the vestibular-cochlear cleavage plane, there is an area of vestibular fibers that are in the cochlear nerve component. An adequate resection of these fibers is crucial to guarantee vertigo freedom during the follow-up. 

Cerebellar retraction and neurovascular manipulation are the most common causes of morbidity related to vestibular nerve sectioning; eliminating the “third hand” from the operative field and using the endoscope guarantees optimal surgical results by taking advantage of its high-magnification power to perform VN at the CPA with a better identification of the nerve cleavage plane, decreasing both operative time and procedural morbidity [22]. 

Multiple reports have described the advantages of endoscopic assistance for VN. King et al. [23] demonstrated that the cleavage plane between the vestibular and the cochlear nerves was identified by the use of a 30-degree endoscope. Miyazaki et al. [24] reported improved visualization of the VII and the VIII nerves using a 30-degree endoscope through the retrosigmoid approach. H. Y. Tan at al. [25] reported that endoscope-assisted microsurgery via a presigmoid retrolabyrinthine approach offers direct access to the CPA, minimizing cerebellar retraction and ensuring hearing preservation. Thanks to the use of the microscopic-endoscopic combined approach (MECA), we did not have major complications. The use of the endoscopic optics at 0°, 30°, and 45° allowed us to perform complete anatomical visualization of the CPA, especially when entering the CPA, without displacement of the cerebellum, which usually reduces the underlying vision. We also conducted a second endoscopic check at the end of the vestibular section. Furthermore, in our experience, the endoscope allowed us to carry out correct visualization of the anatomical relationship between VII and VIII cranial nerves.

Pressure on the sigmoid sinus using the sucker to obtain the sigmoid sinus flattening or mastoidectomy enlargement were performed to improve the working angle, resulting in an increased risk of intraoperative bleeding and hearing loss, due to the drilling of the posterior semicircular canal. The otolaryngologist’s knowledge of the temporal bone anatomy of and the use of the endoscope helped us to avoid these intraoperative complications. The importance of these aspects, in view of a good outcome after VN, is also reported in the literature [16].

We believe that our preliminary experience shows that, in selected case, VN is an effective solution treatment for drug-resistant MD, reaching good functional results, vertigo resolution in the majority of the patients (90% of cases), and an overall low complication rate [26,27]. When vertigo persists, it is possible to perform a targeted MRI focusing on the VIII nerve to demonstrate residual vestibular nerve fibers [1]. In our opinion, scientific research efforts in the future should be directed towards randomized clinical trials that are not currently available in the literature, to the best of our knowledge. Possible applications within the research setting could encompass the use of this surgical technique after medical treatment failure due to its efficacy to control vertigo in patients with MD, confirming previous authors’ findings. There is evidence (Grade B) of the high efficiency of VN, even compared to ITG, in patients with intractable vertigo and serviceable hearing function; moreover, since it does not totally compromise the hearing function, it could replace surgical labyrinthectomy [3].

The life quality of patients with MD is drastically reduced after multiple failed treatments, which was confirmed in our series by the high scores that we obtained from the preoperative questionnaires. The patients displayed improvements in the physical, functional, and emotional scales after VN. Further, the pure-tone bone conduction average showed auditory stability. In our preliminary experience, the experimental details of our surgical approach are the skeletonization of the sigmoid sinus and its retraction. These operative specifics and technical strategies have been based on previous reports, such as in the series of Alonso et al. We feel that it is important to confirm the above-mentioned authors’ findings with regard to the possibility of gaining additional exposure and a wider working angle through the skeletonization of the sigmoid sinus. By doing so, we could overcome the limits of the approach in selected cases and we could potentially suggest technical nuances to our other colleagues for further studies [28].

### Study Limitations

The limitations of the present study are represented by the small sample of selected patients, by the poor reproducibility of the surgical technique in all patients who had different anatomical alterations, by the different preoperative clinical pictures of the patients—which reduced the possibility of homogeneously standardizing the sample from empirically objectivizing postoperative clinical improvement. Further studies will therefore be necessary to address these limitations and provide results that confirm the findings of the present study.

## 5. Conclusions

Selective VN via a presigmoid retrolabyrinthine approach is a safe procedure for intractable vertigo associated with MD, when residual hearing function still exists. This procedure is characterized by a low rate of complications and about 90% vertigo control after surgery. The hearing function damage risk is lower than ITG. The use of the endoscope and intraoperative neuromonitoring guarantees a precise selective VN, saving the cochlear fibers and facial nerve. The approach for VN is a familiar procedure to the otolaryngologist as is lateral skull base anatomy to the neurosurgeon; therefore, the best results can be obtained when utilizing teamwork and a multidisciplinary prospective.

## Data Availability

The original contributions presented in this study are included in this article; further inquiries can be directed to the corresponding authors.
